# The Artificial Intelligence-Assisted Diagnosis of Skeletal Dysplasias in Pediatric Patients: A Comparative Benchmark Study of Large Language Models and a Clinical Expert Group

**DOI:** 10.3390/genes16070762

**Published:** 2025-06-28

**Authors:** Nikola Ilić, Nina Marić, Dimitrije Cvetković, Marko Bogosavljević, Gordana Bukara-Radujković, Jovana Krstić, Zoran Paunović, Ninoslav Begović, Sanja Panić Zarić, Slađana Todorović, Katarina Mitrović, Aleksandar Vlahović, Adrijan Sarajlija

**Affiliations:** 1 Clinical Genetics Outpatient Clinic, Mother and Child Health Care Institute of Serbia “Dr Vukan Cupic”, 11070 Belgrade, Serbia; jovanakrst98@gmail.com (J.K.); adrijans2004@yahoo.com (A.S.); 2 Clinic for Children Diseases, University Clinical Center of the Republic of Srpska, 78000 Banja Luka, Bosnia and Herzegovina; nina.maric@med.unibl.org (N.M.); gordana.bukara-radujkovic@med.unibl.org (G.B.-R.); 3 Medical Faculty, University of Banjaluka, 78000 Banja Luka, Bosnia and Herzegovina; 4 Department of Endocrinology, Mother and Child Health Care Institute of Serbia “Dr Vukan Cupic”, 11070 Belgrade, Serbia; cvetkovic.d@outlook.com (D.C.); sanja.pnc@gmail.com (S.P.Z.); sladjat71@gmail.com (S.T.); mitrovic.sky@gmail.com (K.M.); 5 Department of Orthopedic Surgery and Traumatology, Mother and Child Health Care Institute of Serbia “Dr Vukan Cupic”, 11070 Belgrade, Serbia; marko.bogosavljevic@gmail.com (M.B.); zoran.paunovic@imd.org.rs (Z.P.); ninobego@hotmail.com (N.B.); 6 Department of Surgery, Division of Pediatric Surgery, University of Belgrade, 11000 Belgrade, Serbia; aleksandarvlahovic@yahoo.com; 7 Department of Pediatrics, Faculty of Medicine, University of Belgrade, 11000 Belgrade, Serbia; 8 Department of Plastic and Reconstructive Surgery and Burns, Mother and Child Health Care Institute of Serbia “Dr Vukan Cupic”, 11070 Belgrade, Serbia; 9 Faculty of Medicine, University of Eastern Sarajevo, 73300 Foča, Republic of Srpska, Bosnia and Herzegovina

**Keywords:** skeletal dysplasia, artificial intelligence (AI), large language models (LLMs), pediatric genetics, diagnostic benchmarking, ChatGPT, DeepSeek

## Abstract

Background/Objectives: Skeletal dysplasias are a heterogeneous group of rare genetic disorders with diverse and overlapping clinical presentations, posing diagnostic challenges even for experienced clinicians. With the increasing availability of artificial intelligence (AI) in healthcare, large language models (LLMs) offer a novel opportunity to assist in rare disease diagnostics. This study aimed to compare the diagnostic accuracy of two advanced LLMs, ChatGPT (version GPT-4) and DeepSeek, with that of a clinical expert panel in a cohort of pediatric patients with genetically confirmed skeletal dysplasias. Methods: We designed a prospective vignette-based diagnostic benchmarking study including 45 children with confirmed skeletal dysplasias from two tertiary centers. Both LLMs were prompted to provide primary and differential diagnoses based on standardized clinical case vignettes. Their outputs were compared with those of two human experts (a pediatric endocrinologist and a pediatric orthopedic surgeon), using molecular diagnosis as the gold standard. Results: ChatGPT and DeepSeek achieved a comparable diagnostic top-3 accuracy (62.2% and 64.4%, respectively), with a high intermodel agreement (Cohen’s κ = 0.95). The expert panel outperformed both models (82.2%). While LLMs performed well on more common disorders, they struggled with ultra-rare and multisystemic conditions. In one complex case missed by experts, the DeepSeek model successfully proposed the correct diagnosis. Conclusions: LLMs offer a complementary diagnostic value in skeletal dysplasias, especially in under-resourced medical settings. Their integration as a supportive tool in multidisciplinary diagnostic workflows may enhance early recognition and reduce diagnostic delays in rare disease care.

## 1. Introduction

### 1.1. Context and Diagnostic Challenge

Skeletal dysplasias, also known as osteochondrodysplasias, represent a heterogeneous group of genetic disorders characterized by abnormalities in the development, growth, and maintenance of bone and cartilage tissue [1]. With more than 450 different distinct entities classified by the International Skeletal Dysplasia Society (ISDS), these conditions exhibit substantial genetic and phenotypic variability, often overlapping with other syndromic or metabolic bone diseases [2]. Although each dysplasia is rare, their combined birth prevalence is estimated at approximately 1 in 4000 to 5000 live births, making them a collectively significant entity in pediatric practice [3]. Many skeletal dysplasias are currently considered ultra-rare due to their incidence below 1:50,000 livebirths [4].

From a clinical standpoint, skeletal dysplasias can affect virtually any part of the skeleton: the skull, axial skeleton, and limbs (epiphyses, metaphysis, or growth plates) [5]. These conditions are usually marked by a disproportionate short stature often accompanied by extraskeletal features, such as facial dysmorphism, joint laxity or stiffness, neurodevelopmental delay, hearing loss, visual impairments, cardiac anomalies, and others [6]. This various clinical presentation complicates early recognition, particularly in neonates and infants with suggestive findings [7].

The diagnostic pathway is frequently complex and protracted. It involves a combination of a detailed physical and radiographic assessment, a multidisciplinary evaluation, and targeted or broad-spectrum genetic testing [8]. In many cases, especially those with mild or atypical presentations, the diagnostic process is delayed for months or even years. This delay often leads to further frustration in families, inappropriate treatments, and missed opportunities for genetic counseling or the administration of innovative therapies [9,10].

Furthermore, in healthcare systems with limited access to expert skeletal radiologists or clinical geneticists, recognizing and classifying these rare conditions is particularly challenging [11]. Diagnostic errors are not uncommon, especially in early childhood when features may not yet be fully developed [12]. Even in specialized centers, the clinical overlap between different dysplasias often necessitates a wider approach to genetic testing with varied success. This prompts the need for a more calibrated diagnostic algorithm that can enhance and refine traditional expertise (Table 1) [13,14].

### 1.2. Next-Generation Sequencing: Transformative but Not Definitive

The advent of next-generation sequencing (NGS) has significantly improved the diagnostic yield in monogenic bone diseases (40–70%), with whole-exome sequencing (WES) becoming standard in many tertiary centers [15,16]. However, despite its power, NGS has notable limitations, including the interpretation of the variants of uncertain significance (VUS), incidental findings, and the incomplete detection of pathogenic mutations [17,18,19].

While whole-genome sequencing (WGS) may overcome some limitations of exome-based approaches, such as detecting deep intronic variants, structural rearrangements, or regulatory mutations, it introduces additional challenges related to data interpretation, costs, and clinical implementation [20,21]. An accurate interpretation still requires expert clinical correlation, particularly in pediatric cases where phenotypes may be evolving or atypical [22,23]. In light of these challenges, there is a growing need for decision-support tools that can assist clinicians in integrating molecular data with phenotypic characteristics [22].

### 1.3. Artificial Intelligence in Medical Diagnostics: A New Frontier

AI has recently gained traction in healthcare, offering novel tools for pattern recognition and clinical decision support [24,25]. Its potential lies in improving the diagnostic speed, consistency, and access, particularly in settings where clinical expertise is variable or scarce [26]. Among the most promising innovations are LLMs, such as ChatGPT and DeepSeek Medical, which are capable of processing a complex, unstructured clinical input using natural language [24,27,28]. These models have shown a promising performance in diagnostic tasks, at times rivaling junior medical professionals [27]. However, their real-world application remains largely experimental, especially in rare disease settings where clinical presentations are heterogeneous and complex diagnostic reasoning is required [29].

### 1.4. Study Aim and Diagnostic Benchmarking Framework

Given the diagnostic complexity and clinical variability of hereditary skeletal dysplasias, as well as the limitations of molecular diagnostics, there is a compelling need for novel tools that can augment diagnostic reasoning [3,30]. In this context, the aim of our study was to evaluate the performance of two AI-driven LLMs, ChatGPT (GPT-4) and DeepSeek Medical AI, in interpreting structured clinical vignettes of pediatric patients with genetically confirmed skeletal dysplasias.

Each model was prompted to generate the following: a primary diagnosis, two differential diagnoses, and corresponding confidence levels, based solely on the textual input, mimicking real-world clinical data abstraction.

To benchmark the performance of the AI systems, we compared their diagnostic outputs with those of a human expert panel. This approach enabled a direct head-to-head comparison between artificial and human reasoning in a clinically relevant and phenotype-driven diagnostic context.

To our knowledge, this is the first prospective, vignette-based diagnostic benchmark study applying AI–LLM technology to a cohort of patients with confirmed skeletal dysplasias. The findings of this work may offer valuable insights for the future AI integration into rare disease diagnostics, particularly in settings where subspecialist expertise is limited or delayed [31].

## 2. Materials and Methods

### 2.1. Study Design and Setting

This prospective diagnostic accuracy study was conducted in collaboration between two tertiary-level academic centers: The University Children’s Hospital within the Mother and Child Health Care Institute of Serbia (Belgrade, Serbia) and the University Clinical Center of the Republic of Srpska (Banja Luka, Republic of Srpska, Bosnia and Herzegovina). Both institutions are national referral centers for rare pediatric and genetic diseases. This study received institutional approval and was conducted in accordance with the Declaration of Helsinki and relevant national ethical guidelines for clinical research involving human participants.

### 2.2. Data Curation

The data for this study were obtained from medical records, including both paper and electronic formats, sourced from two centers. Data cleaning involved removing duplicate entries and correcting any obvious data entry errors. A thorough review of documentation was conducted to ensure completeness and accuracy, with no missing critical data identified.

### 2.3. Patient Population and Inclusion Criteria

This study included a total of 45 pediatric patients with genetically confirmed skeletal dysplasias: 25 patients were recruited from the Institute for Mother and Child Health Care of Serbia, and 20 patients were included from the University Clinical Center of the Republic of Srpska.

All patients were initially evaluated by a pediatric clinical geneticist, who assessed the phenotype and indicated NGS as part of diagnostic work-up. NGS was performed using either targeted gene panels related to skeletal dysplasias or WES. Only patients with positive molecular findings were considered eligible for inclusion.

Inclusion criteria were as follows: pediatric age (0–18 years), clinical presentation compatible with a hereditary skeletal disorder, and unequivocally confirmed molecular diagnosis of skeletal dysplasia. Exclusion criteria were as follows: incomplete or insufficient medical documentation for vignette generation and negative or inconclusive genetic results.

### 2.4. Vignette Design and Simulation Protocol

For each patient, a structured clinical vignette was developed using a standardized template to simulate real-world diagnostic challenges. The vignette captured the cross-sectional snapshots, based on the most informative clinical encounters, typically at the time of genetic testing.

Each vignette included the following components:

1. Demographic and Anthropometric Data: Age at presentation, sex, height, weight, and body mass index (BMI), with percentile values based on WHO or CDC standards. When available, additional metrics such as sitting height and head circumference were included.
2. Prenatal and Perinatal History: Key findings such as intrauterine growth restriction (IUGR), oligohydramnios, prenatal suspicion of skeletal anomalies, birth measurements, and perinatal complications.
3. Skeletal and Extraskeletal Findings: A structured description of radiographically and clinically observed features, including short stature patterns (rhizomelic, mesomelic, etc.), joint anomalies, and presence of extraskeletal manifestations (e.g., facial dysmorphism, developmental delay, cardiac or renal anomalies).
4. Radiological Impressions: Summary of key imaging findings, including any radiographs or skeletal surveys interpreted by pediatric radiologists or geneticists, highlighting patterns suggestive of specific dysplasias.
5. Clinical Course: History of fractures, neurodevelopmental milestones, disease progression, or other relevant time-linked features. Vignettes did not describe full longitudinal follow-up but included key evolutional clues when available.
6. Biochemical and Metabolic Parameters: Abnormal laboratory results considered diagnostically relevant (e.g., alkaline phosphatase, calcium/phosphate disturbances, markers of storage disorders).
7. Family History: When available, a brief summary of family history of similar conditions or known genetic diagnoses was included to reflect the clinical reality of hereditary disorders.

To maintain diagnostic neutrality and ensure that LLMs and human raters relied solely on clinical reasoning, genetic results were deliberately withheld during the diagnostic phase.

Each of the 45 vignettes was treated as a standalone diagnostic scenario. Based on estimated prevalence, patients were classified into two categories, rare skeletal dysplasias (n = 21) and ultra-rare skeletal dysplasias (n = 24), defined by the number of reported cases per 50,000 live births, following Orphanet and recent literature benchmarks.

### 2.5. Artificial Intelligence Systems Description

Two state-of-the-art LLMs were employed in this study to simulate autonomous diagnostic reasoning: ChatGPT (GPT-4, OpenAI) is a cutting-edge transformer-based LLM developed by OpenAI, utilizing the GPT-4 architecture. This model has been trained on a mixture of publicly available and licensed datasets. These include internet-scale corpora such as Common Crawl, Wikipedia, open-access books, academic articles, PubMed abstracts, and other publicly available medical literature. Although the full dataset composition is not publicly disclosed, OpenAI confirms that the training data span a wide range of general and technical domains, including biomedical texts, to ensure broad applicability in professional contexts. GPT-4 supports multi-turn dialog, contextual understanding, and dynamic adaptation to complex medical reasoning tasks. In this study, ChatGPT was accessed via the ChatGPT Plus API (2024 version, GPT-4-turbo) and was used to simulate structured diagnostic reasoning based on clinical vignettes. Each interaction involved presenting the model with a case narrative and recording its diagnostic output in a blinded and standardized fashion [27].

DeepSeek is a multilingual LLM developed by DeepSeek-VLLM, optimized for scientific and medical applications [28]. The version used in this study was DeepSeek Medical (2024), which has been pretrained on PubMed articles, case reports, and clinical datasets in both English and Chinese. According to the model’s documentation, this includes English-language web data, GitHub (3.5.0) repositories, Wikipedia, scientific papers, and medical literature. The training also involved domain-specific corpora aimed at enhancing performance in professional and scientific domains, including healthcare and life sciences. As with other LLMs, detailed dataset composition remains proprietary. The model architecture is transformer-based, with support for few-shot and zero-shot learning paradigms [32].

Access to DeepSeek was implemented through the official online web interface under research licensing terms. Prompts identical to those given to ChatGPT were submitted, including structured input and diagnostic format. DeepSeek’s responses were extracted manually due to the limitations of its web interface. No real-time access to EHRs or databases was available to the model.

Both models were engaged under identical conditions and evaluated independently using identical case material.

### 2.6. AI-Based Diagnostic Simulation

Each vignette was submitted to the two AI systems under identical conditions. Both were prompted to generate a primary diagnosis, two alternative differential diagnoses, and a confidence score on a scale of 1–5 (1 being lowest and 5 being highest certainty), for each diagnostic suggestion. The prompts were standardized across all vignettes and both systems to ensure comparability. No additional context, role-playing cues, or guiding frameworks were supplied.

### 2.7. Expert Clinical Panel—Human Comparator

In addition to AI analysis, each of the 45 case vignettes were reviewed independently by a human control group composed of a two-member expert panel: a pediatric endocrinologist with expertise in rare bone diseases and a pediatric orthopedic surgeon with extensive experience in skeletal dysplasias. Panel members were blinded to the genetic results and to each other’s responses. Each expert completed the diagnostic task individually using the same three-step format as the AI (primary diagnosis, two alternatives, confidence scores 1–5). After individual assessments, consensus was reached for each case.

### 2.8. Outcome Measures and Adjudication

The primary outcome was top-3 diagnostic accuracy, defined as the proportion of cases in which the confirmed molecular diagnosis was present among the three suggestions provided by the model or expert panel (primary diagnosis and two alternatives). Subtype discrepancies (e.g., different types of osteogenesis imperfecta) were not considered errors if the general diagnostic category was appropriate.

Secondary outcomes included the following:

− Frequency of correct diagnosis within the top three suggestions;
− Distribution of confidence scores across groups;
− Level of agreement between AI models and human experts.

All results were independently reviewed and adjudicated by a third specialist in medical genetics to ensure consistency and impartial evaluation.

### 2.9. Statistical Analysis

All statistical analyses were performed using IBM SPSS Statistics for Windows, Version 20.0 (IBM Corp., Armonk, NY, USA). Descriptive statistics were calculated for all variables, including means and standard deviations for continuous variables and frequencies with percentages for categorical variables. Bivariate correlations were examined using Pearson’s or Spearman’s method, as appropriate to variable distribution, to explore potential associations between clinical parameters.

To assess the relationship between specific patient characteristics from the vignettes and diagnostic top-3 accuracy, a series of binary logistic regression analyses was conducted. Separate models were created for each evaluator, the human expert group and both LLMs, and for each major category of predictor variables: demographic parameters (age at symptom onset, age at evaluation, and sex), anthropometric characteristics (height Z-score, type of body disproportion, and presence of macrocephaly), and clinical “red flags” (history of pathological fractures, biochemical abnormalities, intellectual disability, vision impairment, and hearing loss).

The dependent variable in all regression models was diagnostic correctness (1 = correct diagnosis, 0 = incorrect diagnosis). Odds ratios (ORs) with corresponding 95% confidence intervals (CIs) were reported to quantify the strength of association between predictors and diagnostic outcome. Given the relatively small cohort size (n = 45), *p*-values < 0.10 were interpreted as indicative of potentially meaningful trends.

## 3. Results

### 3.1. Cohort Characteristics and Clinical Parameters

This study included 45 pediatric patients with genetically confirmed skeletal dysplasias. The cohort was assembled from two large tertiary clinical centers in the Western Balkans: 25 patients were evaluated at the Institute for Mother and Child Healthcare of Serbia and 20 patients at the University Clinical Center of the Republic of Srpska. All 45 patients underwent molecular genetic testing (WES in 27, targeted NGS panel in 12, and 6 single gene analyses), which confirmed a molecular diagnosis consistent with a recognized skeletal dysplasia (Table S1).

The age range spanned from neonatal to adolescent (1 month to 17 years), with a slight male predominance in the cohort (26 males, 19 females). The median age at the time of the clinical evaluation was 5 years, (SD 5.28; range: 0.25–18.0 years), while the median age at the symptom onset was significantly earlier, at 1.2 years (SD 2.39). Patients exhibited a wide range of growth impairments, with a mean height Z-score of −2.29 (SD 2.50), reflecting a population with a pronounced short stature. The most extreme Z-score recorded was −8.34, while the maximum was +2.39. Most patients had a symmetric short stature (60.0%), while 31.1% exhibited a shortening of the limbs, and 8.9% had short trunks. Macrocephaly was documented in 26.6% of the cohort. In 26.7% of patients there was a history of repeated bone fractures.

Developmental delays were present in 42.2% of cases for motor milestones and in 17.8% for intellectual functioning. Neurological abnormalities were relatively uncommon (13.3%), while hearing and visual impairments were each observed in 13.3% and 20.0% of patients, respectively. Biochemical abnormalities that included alkaline phosphatase, electrolyte, hormonal and vitamin D disturbances, and urinary glycosaminoglycan (GAG) elevations were recorded in 42.2% of cases.

### 3.2. Diagnostic Spectrum and Distribution

This cohort demonstrated a high level of diagnostic heterogeneity, with representations from both relatively common rare skeletal dysplasias and multiple ultra-rare or syndromic entities. The most commonly diagnosed conditions in the cohort were osteogenesis imperfecta (OI) and achondroplasia. Thirteen distinct genetic diagnoses were identified in single patients only (Table 2).

### 3.3. Diagnostic Performance of AI Models

ChatGPT achieved 28 correct diagnoses (62.2%) out of 45, while DeepSeek correctly identified 29 cases (64.4%). Both models demonstrated comparable performances, with DeepSeek slightly outperforming ChatGPT. The majority of errors were observed in cases with ultra-rare diagnoses or syndromic phenotypes with overlapping features. ChatGPT gave correct diagnoses as its primary in 55.6%, as a second alternative in 2.2%, and as a third alternative in 4.4%. DeepSeek achieved a 57.8% primary diagnose correctness and 6.7% partial correctness (Table 3).

The intermodel agreement (Cohen’s kappa) between ChatGPT and DeepSeek AI regarding diagnostic accuracy is 0.95, indicating an almost perfect level of agreement between the two models.

### 3.4. Additional Statistical Findings

A two-sided McNemar test was used to assess the diagnostic disagreement between the two AI models across the entire cohort (n = 45). The analysis yielded a *p*-value of 1.000, indicating no statistically significant difference in the diagnostic accuracy between ChatGPT and DeepSeek when operating under identical prompt conditions.

The LLMs’ top-3 accuracy varied notably across more frequent diagnostic entities: OI was correctly classified in 11 of 12 cases (91.6%), achondroplasia was correctly identified in 5 of 6 cases (83.3%)**,** and hypophosphatemic rickets was correctly diagnosed in 2 of 3 patients (66.7%). In contrast, several ultra-rare disorders, such as CODAS syndrome, MPS VI, and Alpha Mannosidosis, were consistently missed by both models.

Among rare disorders (n = 21), ChatGPT achieved a top-3 accuracy rate of 85.7%, compared to 80.9% for DeepSeek. This difference was not statistically significant (McNemar’s test, *p* = 0.25). In the subgroup of ultra-rare disorders (n = 24), the top-3 accuracy dropped to 41.7% for ChatGPT and 50.0% for DeepSeek, with no statistically significant difference observed (McNemar’s test, *p* = 1.0) (Table 4).

### 3.5. Performance of Human Experts and Comparison with AI Models

To benchmark the AI performance against the clinical expertise, the human expert panel achieved a diagnostic top-3 accuracy of 37/45 (82.2%), significantly surpassing the top-3 accuracy of both LLMs in terms of the primary diagnosis (chi square *p* < 0.001). In 8 of 45 cases (17.8%), experts failed to provide a correct diagnosis. Among these eight challenging cases, DeepSeek correctly identified one of them.

The chi-square analysis revealed a statistically significant association between the correct diagnostic performance of both AI models and the expert panel (*p* < 0.001 for both comparisons). Additionally, a strong association was found between ChatGPT and DeepSeek predictions (*p* < 0.001), confirming consistent diagnostic behavior between the two AI models.

Out of 24 patients with ultra-rare disorders, human experts correctly identified 19 cases (79.2%), while both AI models had markedly lower performances (chi square *p* < 0.001). Conditions such as CODAS syndrome, Dyggve–Melchior–Clausen disease, Ellis–van Creveld syndrome, and Alpha Mannosidosis were frequently missed by AI, especially when key clinical elements were dispersed or subtle.

Human experts were also prone to misclassification in ultra-rare conditions—five out of eight total human errors occurred in this subgroup. In several of these cases, the phenotype included neurologic, craniofacial, or systemic abnormalities that typically require the integration of data beyond skeletal findings, such as a developmental history, biochemical work-up, or positive family history.

The binary logistic regression analysis revealed notable differences in how various clinical features influenced the diagnostic accuracy across the human expert panel and the two AI models. Among anthropometric parameters, the presence of macrocephaly showed a suggestive positive association with diagnostic correctness in the human expert (OR = 4.68, *p* = 0.089), indicating that children with enlarged head circumferences were nearly five times more likely to receive a correct diagnosis. This effect was less pronounced and not statistically significant in either AI model.

Among clinical “red flag” variables, a history of bone fractures was the strongest positive predictor of diagnostic success in the human expert model (OR = 17.5, *p* = 0.032); DeepSeek AI also showed a moderate association with fractures (OR = 6.6, *p* = 0.052), whereas ChatGPT demonstrated minimal responsiveness to this cue (OR = 1.9, *p* = 0.40). Biochemical abnormalities were another strong signal for the physicians (OR = 41.7, *p* = 0.074), and to a lesser extent for DeepSeek (OR = 5.5, *p* = 0.079), again with only a modest effect in ChatGPT (Table 5).

In contrast, the presence of intellectual disability negatively impacted the diagnostic accuracy across all models (both AI and the human expert panel), with the strongest effect seen in the human experts (OR = 0.03, *p* = 0.022), highlighting the diagnostic complexity introduced by overlapping neurodevelopmental features. Vision problems also showed a consistent trend toward reducing the diagnostic correctness in all evaluators, although not reaching statistical significance (Table 6).

An analysis of diagnostic confidence levels revealed a consistent trend across all evaluators—higher confidence scores were associated with correct diagnoses. When diagnoses were accurate, the average confidence levels were 4.77 for ChatGPT, 4.83 for DeepSeek, and 4.88 for the expert group. In contrast, incorrect diagnoses were accompanied by lower average confidence scores—4.03 for ChatGPT, 4.45 for DeepSeek, and 4.63 for the experts (Table 7).

Higher diagnostic confidence scores were significantly associated with correct diagnoses for both ChatGPT (*p* = 0.0014) and DeepSeek (*p* = 0.046) but not for the human expert group (*p* = 0.13).

## 4. Discussion

### 4.1. General Diagnostic Performance of LLMs Versus Human Expert Group

This study provides a comparative evaluation of LLMs and a human expert panel in the diagnosis of genetically confirmed skeletal dysplasias in a pediatric cohort. The cohort encompassed a wide clinical and genetic spectrum, including more frequent skeletal dysplasias and a multitude of ultra-rare entities. This diagnostic heterogeneity, together with the broad variation in the age of onset, growth patterns, and systemic involvement, reflects the real-world complexity of skeletal dysplasia diagnostics [3].

Key findings show that while both LLMs, ChatGPT and DeepSeek, achieved a moderate diagnostic top-3 accuracy (62.2% and 64.4%, respectively), they were significantly outperformed by the human expert group (82.2%). Nonetheless, the two LLMs exhibited remarkably similar diagnostic behavior, indicating a convergence in reasoning strategies.

### 4.2. Impact of Disorder Prevalence on AI Performance

The diagnostic performance of AI models was notably influenced by the disorder prevalence. For the more frequent rare conditions, such as OI and achondroplasia, LLMs performed robustly. In contrast, the diagnostic yield for ultra-rare conditions dropped markedly. This discrepancy presents a correct depiction of the current limitations of LLMs in the assessment of the subtle, multisystemic presentations without strong phenotypic markers [33,34].

Among the 21 patients with more common skeletal dysplasias, both ChatGPT and DeepSeek achieved a relatively high diagnostic top-3 accuracy (85.7% and 80.9%, respectively). This fact depicts the strengths of LLMs in pattern recognition when exposed to well-characterized syndromes with distinct skeletal characteristics. In particular, OI was correctly classified in 11 out of 12 cases (91.6%), suggesting that classical radiological and clinical features, such as blue sclerae and recurrent bone fractures, were effectively captured and weighted by the models. Interestingly, ChatGPT demonstrated a slightly higher top-3 accuracy than DeepSeek in the subgroup of rare skeletal disorders, matching the performance of the experienced human expert group. Although the difference did not reach formal statistical significance (due to the limited cohort size), the trend suggests that ChatGPT may possess a greater generalization capacity for well-characterized, pattern-based conditions.

In the group of ultra-rare skeletal dysplasias, the diagnostic performance sharply declined. Both LLMs achieved a markedly lower top-3 accuracy—ChatGPT achieved 41.7% and DeepSeek achieved 50.0%. This drop points out a key limitation of current LLMs. Their diagnostic accuracy appears to depend heavily on how well specific disorders are represented in their training data. When faced with phenotypically heterogeneous, multisystemic conditions, such as CODAS syndrome or Dyggve–Melchior–Clausen disease, both AI systems struggled. These findings align with prior studies reporting a reduced LLM diagnostic confidence in ultra-rare disease cohorts, especially when presentations deviate from classical textbook phenotypes [35,36,37].

### 4.3. Diagnostic Accuracy and Phenotypic Distinctiveness

Another major determinant of the AI model performance in our study was the diagnostic recognizability of the disorder in question. Our regression analysis offers insights into how different evaluators, the human experts group and two LLMs, weigh specific phenotypic, clinical, and diagnostic features when formulating diagnostic impressions.

The human experts showed a significant reliance on clinically striking indicators. The presence of a repeated bone fracture increased the likelihood of a correct diagnosis by more than 17-fold, while abnormal laboratory findings also demonstrated a strong positive association. These findings are confirming the expected behavior of an experienced clinician trained to recognize typical “red flag” patterns [13,30]. It also further reflects the classical heuristic nature of human reasoning and a tendency to rely on cue-based pattern recognition rather than methodically weighing every piece of information [38].

By contrast, ChatGPT exhibited weak and statistically non-significant associations with nearly all clinical features. The modest increase in diagnostic accuracy associated with fractures and biochemical findings suggests that this AI model applies a more evenly distributed attention to input variables, rather than prioritizing specific diagnostic features [39]. This may reflect its general-purpose design, which is not specifically trained for medical diagnostic tasks [27].

The DeepSeek LLM, however, demonstrated an intermediate profile. While its associations were weaker than those of the human expert, DeepSeek responded positively to the bone fracture history and biochemical abnormalities, suggesting the partial mimicry of physician heuristics. This pattern suggests that the model is, to some extent, fine-tuned on biomedical data and partially optimized for clinical reasoning. As a result, it more closely resembles the human logic in the diagnostic process [33].

Interestingly, macrocephaly, as a relatively common phenotypic feature in certain skeletal dysplasias, also showed a trend toward influencing the physician diagnostic accuracy, while having minimal effects in the AI models. This further supports the hypothesis that the human diagnostic process may be shaped by the subconscious weighting of prominent clinical features, particularly when tied to well-known syndromes [40].

Intellectual disability, on the other hand, was negatively associated with the diagnostic correctness across all evaluators, most prominently in the human expert. This finding reflects the additional complexity of the neurodevelopmental features and their ability to mask or overshadow skeletal elements and suggests that both humans and LLMs are susceptible to diagnostic dilution in such settings [7,30].

Taken together, the results of the regression analysis illustrate the differing cognitive architectures of human and AI diagnostic procedures. The physician operates with a high sensitivity to certain phenotypic characteristics but may overlook atypical or mixed presentations [30]. On the other hand, ChatGPT offers a broad but shallower diagnostic lens, while DeepSeek appears to represent a hybrid model, mimicking the human expert behavior for the classical features of skeletal dysplasia but still limited in recognizing the full clinical spectrum [27,33,35].

### 4.4. Diagnostic Confidence and Interpretability of AI Decisions

The observed association between the diagnostic accuracy and confidence levels of LLMs suggests that confidence may partially reflect the internal coherence or recognizability of the phenotypic pattern being assessed. This association was statistically significant for both AI models, indicating that their confidence scores may serve as a useful indicator for diagnostic reliability. In contrast, among human experts, this trend was not statistically significant, possibly reflecting a more uniformly high level of self-assurance regardless of diagnostic correctness. Importantly, the presence of a moderate to high confidence even in incorrect assessments across all groups highlights the limitations of relying solely on confidence as a marker of diagnostic accuracy [30,33].

### 4.5. Strengths and Limitations of LLMs in Diagnostically Challenging Cases

Despite being outperformed overall by the human expert panel (82.2% versus ~63% top-3 accuracy), both AI models demonstrated a reliable diagnostic utility in cases of skeletal dysplasias with clearly recognizable phenotypic characteristics.

Notably, in 8 out of 45 patients (17.8%), neither the pediatric endocrinologist nor the orthopedic surgeon identified the correct diagnosis. In one of these diagnostically unresolved cases in the expert group, DeepSeek successfully identified the correct condition. In this patient with the diagnosis of Acrofacial Dysostosis, the LLM may have drawn on less obvious associations between diverse clinical features and less conventional diagnostic pathways. This finding may be particularly relevant in settings where clinical suspicion is diluted by atypical findings or when physicians lack subspecialty experience with rare syndromes [11,30,41].

Nonetheless, this complementary role of LLMs should not be overstated. In the majority of more challenging cases, expert clinicians provided the correct diagnosis when AI models failed. This confirms that deeply empirical clinical reasoning; the integration of the physical, radiological, and laboratory findings; and the recognition of subtle features remain uniquely human strengths [9,14,40].

An analysis of AI model errors reveals that diagnostic failures were disproportionately concentrated among ultra-rare or phenotypically ambiguous disorders. Conditions such as CODAS syndrome, Ellis–van Creveld syndrome, Dyggve–Melchior–Clausen disease, and alpha mannosidosis were consistently misdiagnosed by both ChatGPT and DeepSeek, despite the correctly formatted prompts and access to comprehensive phenotypic descriptors. These disorders often manifest with multisystem involvement, subtle craniofacial features, or progressive characteristics that evolve over time. These elements, although well documented, have not been weighted appropriately in the LLM inference.

Another common pattern in misclassified cases was the presence of overlapping phenotypic features. For example, short statures, skeletal deformities, and developmental delay are classical features of numerous dysplasias, yet the subtle distinctions (e.g., metaphyseal irregularities vs. epiphyseal dysplasia) were frequently underappreciated by the AI systems. This suggests a current limitation in the recognition of fine diagnostic fingerprints and a tendency toward relaying on broad categorical similarities [36,42].

### 4.6. LLM Concordance and the Value of Multi-Model Usage

The exceptionally high intermodal agreement observed between ChatGPT and DeepSeek suggests that, when prompted under standardized conditions, current LLMs often lead to very similar diagnostic reasoning pathways. This similarity likely reflects the shared exposure to overlapping biomedical data from the available literature, clinical guidelines, and online case reports during the model pretraining [40]. From a clinical informatics perspective, such consistency is reassuring. It implies replicability and predictability, which are key features for any decision-support tool considered for integration into the diagnostic methodology (Table 8) [34,43].

However, this convergence also raises questions about the added value of deploying multiple LLMs in parallel for diagnostic usage. If outputs are highly comparable, model plurality may offer diminishing returns unless model architectures or training strategies are deliberately diversified [44]. In our dataset, cases where the two models disagreed were rare, but when they did occur, the disagreement did not systematically favor one model over the other. The lack of systematic disagreement supports the notion that neither LLM exhibits a clear diagnostic advantage over the other.

Clinically, this supports the notion that a single well-calibrated LLM system may be sufficient in many real-world medical scenarios [40]. Nevertheless, in borderline or atypical cases, the sequential use of multiple models could serve as a form of an algorithmic second opinion [44].

DeepSeek and ChatGPT occasionally failed on different cases, despite their high overall agreement. This may reflect subtle differences in model architecture, targeted data training, or attention weighting [45]. For instance, DeepSeek appeared slightly to be more successful in dealing with the complex multisystem input (e.g., mucopolysaccharidoses with cognitive impairment), while ChatGPT showed a stronger performance in cases with classical phenotypic patterns. These discrepancies, while minor, underline the importance of understanding the “black box” nature of LLMs when interpreting outputs in a clinical setting [27,34,39].

Interestingly, the complementary nature of the human expert and AI reasoning reveals a potential for synergy. While human experts were superior at integrating contextual clinical information, AI models occasionally identified rare conditions by leveraging text-based similarity patterns. This suggests that an optimal diagnostic performance may emerge from a hybrid approach—combining the structured reasoning of clinicians with the expansive pattern recognition capabilities of LLMs (Table 9) [34,45].

Future implementations of AI in rare disease diagnostics should therefore focus on collaborative interfaces that enhance, rather than replace, clinical expertise. Models capable of dynamically learning from expert feedback or highlighting diagnostically uncertain cases for a secondary review could serve as powerful next-generation tools in modern hospital settings [42,43,46].

Several limitations of this study must be acknowledged when interpreting the findings. First, although our cohort is enriched for diagnostic heterogeneity, its modest size limits the statistical power to detect subtle differences in performances across models or clinical subgroups. Furthermore, while all diagnoses were genetically confirmed, the phenotypic data provided to AI models were based on pre-structured clinical vignettes rather than full medical records. Although this standardization allowed for a fair comparison across systems, it may have restricted the models’ access to certain contextual details that would otherwise be informative in the process of clinical reasoning.

Another limitation lies in the prompting strategy: both ChatGPT and DeepSeek were tested using identical, human-curated inputs designed to reflect real-world case presentations. While this ensured equality in the model evaluation, it does not fully reflect how LLM tools might be used in practice. In the everyday clinical scenarios, the input quality can vary widely depending on the user’s expertise, documentation habits, and clinical setting.

Another important limitation is the inherent constraint of the vignette-based study design, which did not allow for dynamic interactions. In real-world clinical settings, both physicians and LLMs would likely benefit from the ability to ask clarifying questions, gather additional history, or explore ambiguous findings further—factors that could substantially improve the diagnostic accuracy in practice.

This study also involved only two human experts, each from distinct subspecialties. While this offered a valuable comparative benchmark, the absence of a broader multidisciplinary panel may limit the ability to generalize the accuracy of the expert estimates. Moreover, the diagnostic adjudication was based on genetic results as the gold standard, which may not account for the phenotypic variability or evolving reclassifications of variants over time.

Finally, it is important to note that both AI models are proprietary and may differ in their update cycles, transparency, and underlying data sources. Their performance in this study cannot be assumed to generalize to other LLMs without further validation.

## 5. Conclusions

Our study demonstrates that while human experts currently outperform AI models in the diagnosis of pediatric skeletal dysplasias, LLMs such as ChatGPT and DeepSeek show promising diagnostic capabilities, particularly in well-defined disorders. Their limitations in ultra-rare or phenotypically complex cases are pointing out the importance of clinical expertise. However, the observed complementarity between human expert and AI diagnostic patterns suggests that hybrid models combining structured clinical reasoning with data-driven pattern recognition could enhance diagnostic accuracy. Future implementations should prioritize integrative systems that allow dynamic interactions between human experts and AI, ultimately facilitating more timely and accurate diagnostics in rare disease settings.

## Figures and Tables

**Table 1:** Challenges in the diagnosis of pediatric skeletal dysplasia.

Diagnostic Challenge	Explanation/Impact
High Genetic Heterogeneity	>450 disorders with diverse inheritance and mutational spectrum
Phenotypic Overlap	Many dysplasias share clinical and radiographic features
Age-Dependent Expression	Some features (e.g., metaphyseal changes) appear later in life
Radiological Expertise Often Lacking	Interpretation errors common in early infancy
Limited Access to Clinical Geneticists	Especially in low-resource or regional healthcare settings
Delayed Molecular Testing and Interpretation	NGS not always available or rapidly interpreted
Evolving or Atypical Presentations	Non-classic phenotypes often lead to misdiagnosis or delayed diagnosis

NGS—next-generation sequencing.

**Table 2:** Distribution of diagnosed conditions by genetic etiology.

Genetic Diagnosis	Number of Cases (n)
Osteogenesis Imperfecta (Types I, III, IV)	12
Achondroplasia	6
Hypophosphatemic Rickets	4
Mucopolysaccharidosis IVA (Morquio Syndrome)	4
Metaphyseal Chondrodysplasia (Schmid Type)	2
CODAS	2
Larsen Syndrome	2
Others *	13
Total	45

* Others include the following: Stickler syndrome, Hunter syndrome, Alpha Mannosidosis, Cleidocranial dysplasia, Pseudoachondroplasia, Hypochondroplasia, Ellis–van Creveld syndrome, Freeman–Sheldon syndrome, Acrofacial Dysostosis, ABL1 syndrome, Dyggve–Melchior–Clausen disease, Congenital spondyloepiphyseal dysplasia, and Short-Rib Thoracic Dysplasia type 9 (1 case each).

**Table 3:** Frequency of correct diagnosis within top 3 diagnosis suggestions in LLM.

Evaluator	Correct as Primary Diagnosis	Correct as 2nd or 3rd Alternative Diagnosis	Total Top 3 Accuracy
ChatGPT	25/45 (55.6%)	3/45 (6.6%)	28/45 (62.2%)
DeepSeek	26/45 (57.8%)	3/45 (6.7%)	29/45 (64.4%)

**Table 4:** Diagnostic top-3 accuracy by disorder category.

Skeletal Dysplasia Disorder Rarity Category	ChatGPT	DeepSeek	Expert Panel
Rare Disorders (n = 21)	18/21 (85.7%)	17/21 (80.9%)	18/21 (85.7%)
Ultra-Rare Disorders (n = 24)	10/24 (41.7%)	12/24 (50.0%)	19/24 (79.2%)

**Table 5:** The most common clinical “red flags” in correctly diagnosed cases.

Clinical Feature	Frequency in Correct Diagnoses (n = 37)	% of Correct Diagnoses
Repeated Bone Fractures	14	37.8%
Biochemical Abnormalities	13	35.1%
Limb Shortening	12	32.4%
Macrocephaly	10	27.0%
Facial Dysmorphism	8	21.6%
Motor Developmental Delay	7	18.9%
Vision or Hearing Impairment	4	10.8%

**Table 6:** Predictors of diagnostic success: logistic regression analysis.

Clinical Feature	Expert OR (*p*)	ChatGPT OR (*p*)	DeepSeek OR (*p*)
Repeated Bone Fractures	17.5 (0.032)	1.9 (0.40)	6.6 (0.052)
Macrocephaly	4.68 (0.089)	1.02 (0.97)	1.87 (0.43)
Biochemical Abnormalities	41.7 (0.074)	3.3 (0.15)	5.5 (0.079)
Intellectual Disability	0.03 (0.022)	0.70 (0.65)	0.81 (0.77)
Vision Impairment	0.34 (0.40)	0.52 (0.38)	0.58 (0.42)

**Table 7:** Average confidence scores in correct vs. incorrect diagnoses.

Evaluator	Correct Diagnosis (Avg. Score)	Incorrect Diagnosis (Avg. Score)
ChatGPT	4.77	4.03
DeepSeek	4.83	4.45
Expert Panel	4.88	4.63

**Table 8:** Summary of diagnostic concordance and discordance between evaluators.

Evaluator Pair	Concordant Diagnoses (n)	Discordant Diagnoses (n)	% Concordance
ChatGPT vs. DeepSeek	41	4	91.1%
Expert Panel vs. ChatGPT	28	17	62.2%
Expert Panel vs. DeepSeek	29	16	64.4%

Note: Concordance was defined as matching correct or incorrect primary diagnoses between evaluators.

**Table 9:** Key takeaways for future AI integration in rare disease diagnostics.

Insight	Implication for Practice
LLMs perform well in well-characterized disorders	Use AI to screen for common dysplasias or aid non-specialists
Lower AI top-3 accuracy in ultra-rare or syndromic conditions	Expert review remains crucial for unusual phenotypes
High inter-LLM concordance	A single optimized model may be sufficient in many clinical settings
AI occasionally outperforms experts	Potential as a second opinion or in ambiguous cases
Diagnostic confidence correlates with correctness	Confidence estimates may assist in prioritizing cases for expert review

Note: These strategic recommendations are based on the observed diagnostic behavior of LLMs across 45 rare disease cases of our study.

## Data Availability

The data presented in this study are only available on request from the corresponding author due to privacy or ethical restrictions.

## Supplementary Materials

The following supporting information can be downloaded at https://www.mdpi.com/article/10.3390/genes16070762/s1, Table S1: Comprehensive Phenotypic, Molecular, and AI-Based Diagnostic Data for the Cohort of 45 Pediatric Patients with Skeletal Dysplasia.

## Abbreviations

The following abbreviations are used in this manuscript:

NGS	Next-Generation Sequencing
WES	Whole-Exome Sequencing
VUS	Variants of Uncertain Significance
WGS	Linear Dichroism
OI	Osteogenesis Imperfecta
AI	Artificial Intelligence
LLMs	Large Language Models
USMLE	United States Medical Licensing Examination
